# Acidity and hypoxia of tumor microenvironment, a positive interplay in extracellular vesicle release by tumor cells

**DOI:** 10.1007/s13402-024-00969-z

**Published:** 2024-07-18

**Authors:** Silvia Peppicelli, Lido Calorini, Francesca Bianchini, Laura Papucci, Lucia Magnelli, Elena Andreucci

**Affiliations:** Department of Experimental and Clinical Biomedical Sciences “Mario Serio”, University of Florence, Florence, 50134 Italy

**Keywords:** Tumor microenvironment, Hypoxia, Acidosis, Extracellular vesicles, Cancer progression

## Abstract

The complex and continuously evolving features of the tumor microenvironment, varying between tumor histotypes, are characterized by the presence of host cells and tumor cells embedded in a milieu shaped by hypoxia and low pH, resulting from the frequent imbalance between vascularity and tumor cell proliferation. These microenvironmental metabolic stressors play a crucial role in remodeling host cells and tumor cells, contributing to the stimulation of cancer cell heterogeneity, clonal evolution, and multidrug resistance, ultimately leading to progression and metastasis. The extracellular vesicles (EVs), membrane-enclosed structures released into the extracellular milieu by tumor/host cells, are now recognized as critical drivers in the complex intercellular communication between tumor cells and the local cellular components in a hypoxic/acidic microenvironment. Understanding the intricate molecular mechanisms governing the interactions between tumor and host cells within a hypoxic and acidic microenvironment, triggered by the release of EVs, could pave the way for innovative strategies to disrupt the complex interplay of cancer cells with their microenvironment. This approach may contribute to the development of an efficient and safe therapeutic strategy to combat cancer progression. Therefore, we review the major findings on the release of EVs in a hypoxic/acidic tumor microenvironment to appreciate their role in tumor progression toward metastatic disease.

## Introduction

Unlike normal cells of tissues where each cell phenotype is compartmentalized, solid tumors are characterized by intra-tumoral heterogeneity largely depending on hypoxia and acidity of their microenvironment (tumor microenvironment, TME). Indeed, solid tumors are frequently characterized by hypoxia generated by the unlimited proliferation of tumor cells exceeding the ability of the host blood vessels to satisfy oxygen demand. Thus, low oxygen tension is one of the inevitable conditions of tumors, and the regions with low oxygen tension are defined as hypoxic.

**Fig. 1:**
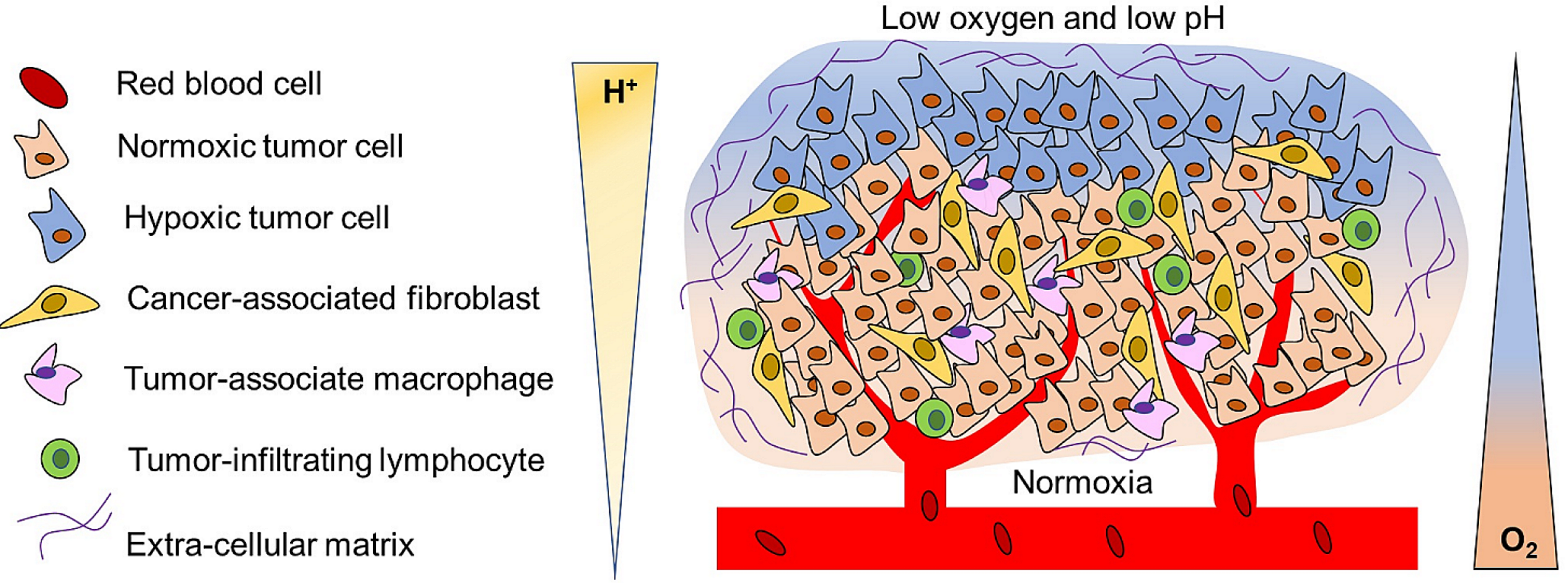
The tumor microenvironment (TME) is represented by a complex network of cellular and non-cellular components surrounding the tumor. It is composed of cancer-associated fibroblasts, adipocytes, endothelial cells, tumor-associated macrophages, and other immune cells, as well as the extracellular matrix, tumor vasculature, and signaling molecules. Moreover, TME is often characterized by acidosis and hypoxia

Angiogenesis, a key step in tumorigenesis, necessary for tumor cell survival and growth, is often a dysregulated phenomenon in tumors [1]. Abnormal and dysfunctional vascular structures, characterized by numerous fenestrations, an often-absent basement membrane, and high vascular permeability, highly contribute to hypoxia. Blood flow in the new tumor vessels is chaotic and irregular due to a lot of redundant branching and shunts. Considering that the limit of oxygen diffusion is around 100–200 μm, it is frequent that most solid tumor regions experience hypoxia [1], which can be an acute stimulus, when tumor cells exposed to hypoxia are promptly re-oxygenated, or a chronic stimulus when tumor cells are exposed to a prolonged state of hypoxia before undergoing death or re-oxygenation. Re-oxygenation results in free radical production, the so-called “reperfusion injury”, exerting further promoting alterations on the genotype of cancer cells. Thus, tumor cells need to adapt to these dynamic changes reprogramming their phenotype. Crucial adaptation to low oxygen (1.5–0.5% oxygen) is the metabolic switch from aerobic glycolysis (the so-called “Warburg effect”) to anaerobic glycolysis. The enhanced glucose utilization by tumor cells even in well-oxygenated areas is used in clinics, and the ^18^F-fluorodeoxyglucose (FDG)-positron emission tomography (PET) imaging plays a role in the management of patients with suspected tumor disease. A change toward anaerobic glycolysis affects the proliferation of tumor cells reducing the possibility of using the intermediates of the glycolytic pathway for anabolic reactions [2], causing an excess lactate/proton extrusion, due to the restriction of entry of metabolites into the mitochondria. Considering that most tumor regions, including those hypoxic areas, are characterized by a high interstitial fluid pressure due to the leakiness from the defective tumor vasculature and the reduced cleared activity of intratumoral lymphatics, it is tough for the acidic products of glycolysis to be drained away. Thus, extracellular space becomes acidic, and the intracellular pH of cancer cells remains neutral or weakly alkaline, suitable for proliferation. In vitro and in vivo studies showed that tumor cells have an intracellular pH (pHi) ranging from 7.12 to 7.56 (pHi of normal cells: 6.99–7.20), and an extracellular pH (pHe) from 6.2 to 6.9 (pHe of normal extracellular space is 7.3–7.4), revealing a reversed pH gradient across the plasma membrane of tumor cells [3, 4]. The maintenance of this gradient is due to the capacity of several transport proteins to release protons (H^+^) and acidify the extracellular environment [4–6]. It would be expected that low extracellular pH and hypoxia co-localize within low blood flow regions, instead low pH was also found in well-oxygenated areas, the so-called “normoxic acidosis” [7]. There are at least two main sources of H^+^ ions in tumors, lactic and carbonic acid, with the former resulting from anaerobic glycolysis and the latter from the conversion of CO_2_ and H_2_O to HCO_3_^−^ and H^+^ by carbonic anhydrase (CA), a family of zinc metalloprotein enzyme [8]. Acid/base homeostasis in cancer cells is regulated by the interplay between CAs and various transport proteins, often forming a protein complex indicated as ‘transport metabolon’ [9]. Thus, hypoxia and acidosis are now considered among the main physicochemical characteristics of the TME (Fig. 1).

Here, we will focus on a particular and not yet completely disclosed aspect of tumor biology influenced by hypoxia and acidosis of TME which is the tumor/stroma cell ability to release extracellular vesicles (EVs). There are some indications that microenvironmental acidity and hypoxia may influence the amount and content of EVs produced by tumors. EVs are membrane-enclosed structures released into the extracellular milieu by tumor/host cells involved in the cross-talk within tumor-tumor/tumor-stromal subpopulations. Now it is known that EVs, rather than serving to get rid of unwanted material [10], mediate fine local and distant intercellular communication under physiological and pathological conditions [11, 12]. According to the guidelines by the International Society of Extracellular Vesicles (ISEV) drawn up in 2018 in the Minimal Information for Studies of Extracellular Vesicles 2018 (MISEV2018) the term ‘extracellular vesicle’ refers to particles that are naturally released from cells, enclosed by a lipid bilayer, and unable to replicate because they lack a functional nucleus. Under the umbrella term EV, there are included exosomes and ectosomes (also called microparticles or microvesicles), which differ from each other in size and subcellular origin. The terms “exosomes” and “ectosomes” are now discouraged unless their subcellular origin can be demonstrated [13, 14] in favor of operational terms based on: size (“small EVs” – sEVs; < 100 nm or < 200 nm), and “medium/large EVs” -m/lEVs; > 200 nm for medium/large), density (low, middle, high), biochemical composition (CD63+/CD81+- EVs, Annexin A5-stained EVs, etc.), or conditions of cells of origin (podocyte EVs, hypoxic EVs, large oncosomes, apoptotic bodies) [13]. The term “extracellular particle” (EP) may be more appropriate in case EVs cannot be identified according to the minimal requirements of the MISEV2018 guidelines [15].

EVs can be isolated from culture media, biological fluids, and tissues via centrifugation, gradient, chromatography, and immunoaffinity methods. Upon isolation, EVs should be characterized based on their protein content and tested for at least one marker for each of the following categories:

- Transmembrane or GPI-anchored proteins associated with the plasma membrane and/or endosomes (e.g., tetraspanins, MHC class I and II, integrins, syndecans).
- Cytosolic proteins (e.g., ESCRT-I/II/III, ALIX, flotillins-1 and 2, caveolins, annexins).
- Major components of non-EV co-isolated structures (e.g., lipoproteins, apolipoproteins A1/2 and B, albumin).

EVs contain a wide variety of bioactive proteins [16], RNA transcripts, micro-RNAs (miRNAs) [17], and even fragments of DNA, which can be transferred to other cancer cells, as well as normal host cells of TME, causing the recipient cells to undergo phenotypic changes promoting progression [18]. Further, the amount of EV release varies depending on the stage, proliferation, and response to therapies of tumor cells, raising the possibility that EVs can be also used for diagnostic purposes [19]. Thus, it is needed to study the release of EVs by cancer/host cells in a hypoxic and acidic microenvironment to have a more complete scenario of tumor cell adaptation.

## Extracellular vesicle biogenesis and function

sEV length 30–100 nm in diameter deriving from the inward budding of the limiting membrane of early endosomes, which mature into multivesicular bodies (MVBs) during the process [10, 20, 21]. MVBs are eventually either sent to lysosomal degradation or fused with the cell’s plasma membrane to release its content, including sEVs, into the extracellular space [20, 22, 23]. The presence of cholesterol seems to dictate the MVB fate [24]. Although the mechanism is not yet fully understood, MVB formation is stimulated by growth factors, and the cell adjusts its sEV production according to its needs [25, 26]. Being MVB regulated by the Endosomal Sorting Complexes Required for the Transport (ESCRT) pathway [27], all the proteins involved in that process (i.e., Alix, TSG101, HSC70, and HSP90β) are expected to be found generally in sEVs regardless of the cell type from which they originate [28, 29]. Traditional surface sEV biomarkers - exploited for isolation - are the tetraspanins CD9, CD63, and CD81 [30], but their heterogeneous presence and abundance highlight the limitations of using these tetraspanins as markers for sEVs derived from different cell types [31]. On the other hand, Syntenin-1 has been proposed as a putative universal sEV marker, due to its role in their biogenesis, and especially because it was found in sEVs across different species and biofluids [31–33]. An alternative ESCRT-independent mechanism of sEV release has been identified as well [34], and it is thought to be dependent on the sphingomyelinase enzyme [35–37].

m/lEVs are medium-large EVs with a 150–1000 nm diameter in length. They are bud away from the plasma membrane with a mechanism that is still not completely known that is thought to require cytoskeleton components (i.e., actin and microtubules), molecular motors (i.e., kinesins and myosins), and fusion machinery (SNAREs and tethering factors) [38]. The number of m/lEVs produced depends on the donor cell’s physiological state and microenvironment [39]. As a result of their biogenesis, m/lEVs show specific proteins regardless of the cell type they derive from [40]. Being MVs originating by outward budding of the cell’s plasma membrane, m/lEVs contain mainly cytosolic and plasma membrane-associated proteins, especially proteins known to cluster at the plasma membrane surface, such as tetraspanins [41, 42], such as CD63, CD9, and CD81, commonly found in sEVs and apoptotic bodies as well [43, 44]. Other proteins commonly identified in m/lEVs include cytoskeletal proteins, heat shock proteins, integrins, and proteins containing post-translational modifications, such as glycosylation and phosphorylation [45, 46]. A specific marker of m/lEVs distinct from sEVs has been identified as Annexin A1 [47].

Despite the difficulties in identifying specific protein markers of the different subclasses of EVs, they show different protein profiles due to their different routes of formation [43, 47, 48]. For instance, actinin-4 and mitofilin have been proposed as specific markers of mEVs and lEVs– absent instead in sEVs -, while syntenin-1, TSG101, ADAM10, and EHD4 are only present in the sEVs, with syntenin-1 and TSG101 being specific of sEVs [49]. Nevertheless, a substantial overlap of protein profiles is often observed [10, 50].

**Fig. 2:**
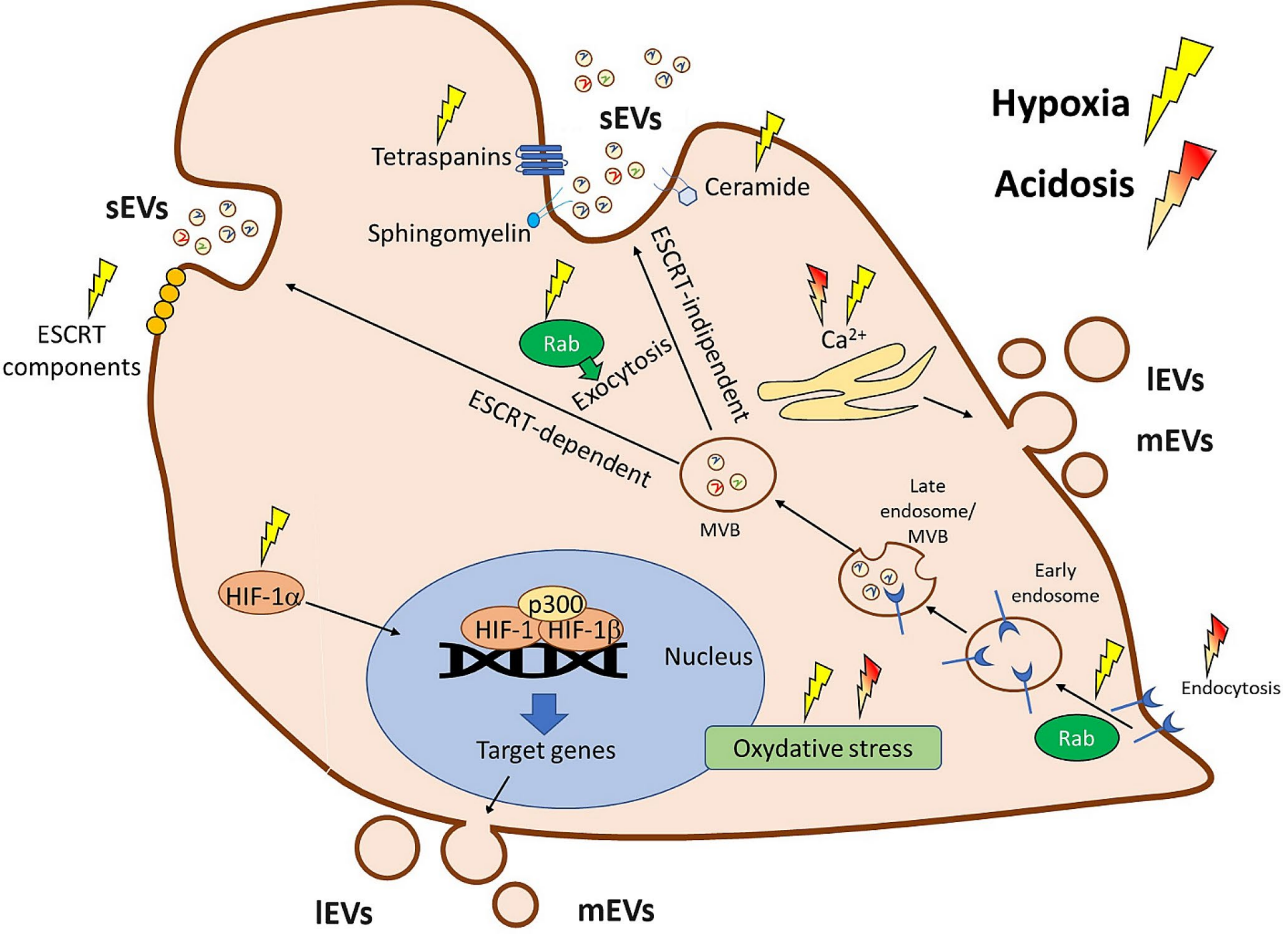
Schematic representation of the regulation of extracellular vesicle biogenesis by hypoxia and acidosis. *sEVs* small Extracellular Vesicles; *mEV* medium Extracellular Vesicles; *lEV* large Extracellular Vesicles; *MVB* multi-vesicular bodies. There are several described mechanisms involved in the higher EV secretion under hypoxia and acidosis. HIF1α signaling, induced in hypoxic conditions, regulates the expression of numerous genes involved in cellular adaptation to low oxygen levels, including genes associated with EV biogenesis, cargo loading, and release. Hypoxia can also lead to the upregulation of Rab, which is essential for the formation and trafficking of MVB, the precursors of smallEVs. Moreover, hypoxia modulates the synthesis of ceramide from sphingomyelin, the regulation of ESCRT machinery, and tetraspanins, and like acidosis, influences the intracellular calcium (Ca^2+^) levels and oxidative stress

There are several mechanisms through which EVs and their contents can be delivered to recipient cells. EVs can dock at the plasma membrane of a target cell [21, 51], and aggregated EVs may potentially fuse directly with the plasma membrane of the recipient cell [21, 51]. Furthermore, pooled EVs can be picked up by processes such as phagocytosis, macropinocytosis, lipid raft-mediated endocytosis, clathrin-mediated endocytosis, and caveolin-mediated endocytosis (Fig. 2) [21, 51, 52]. The moment they are endocytosed, EVs can be targeted to lysosomes for degradation [21, 51] or fuse with the delimiting membrane of an endocytic compartment, thus permitting the discharge of EV contents into the cytosol of the recipient cells [21, 51]. EVs transport bioactive molecular compounds, which may influence the features and phenotypes of recipient cells by affecting gene expression via de novo translation, post-translational modification of target mRNAs [53, 54], or triggering multiple signaling pathways [51, 54].

Overall, the different EV formation pathways and diverse mechanisms of content delivery, underscore the complexity and versatility of EV-mediated cellular communication.

At this point, to comprehend the significance of EV release by tumor cells, it is crucial to study how these vesicles are released under different physiochemical conditions of the TME, such as hypoxia and acidosis, which tumor cells adapt to during disease progression.

### Effects of hypoxic EVs

Tumor hypoxia has long been recognized to be associated with poor prognosis and resistance to therapy, including radiation therapy [55]. Indeed, longer exposure to hypoxia is associated with a high frequency of DNA breaks, and accumulation of DNA replication errors since hypoxia hampers DNA repair systems including homologous recombination and mismatch repair, potentially leading to genetic instability and mutagenesis [56, 57]. Hypoxic response promotes all steps of blood vessel formation [58], but also vasculogenic mimicry in various tumors thanks to HIF family member activity [59]. Furthermore, the hypoxic microenvironment can induce tumor cells to alter the expression of epithelial-mesenchymal transition (EMT) markers and increase the production of matrix metalloproteinases (MMPs) promoting invasion and metastasis [8, 60].

Several findings have reported that microenvironmental hypoxia is capable of modifying the amount and content of EVs released by tumors [61–65]. King et al. [62], and a few years later Wang et al. [61], observed that exposure of breast cancer cells to hypoxia conditions leads to the production of a higher number of EVs. Similarly, the hypoxia-mediated increase in sEV number was reported in different ovarian cancer cell lines [66], in human and mouse glioblastoma cell lines [67], in pancreatic cancer cell lines [68], and in colon cancer cell lines [69]. However, the increased ability to produce EV under hypoxia is not an exclusive phenomenon of tumor cells, but it was described also in MSC [70], adipocytes [71], cancer-associated fibroblasts (CAFs) [72, 73], and tumor-associated macrophages (TAMs) [74].

**Fig. 3:**
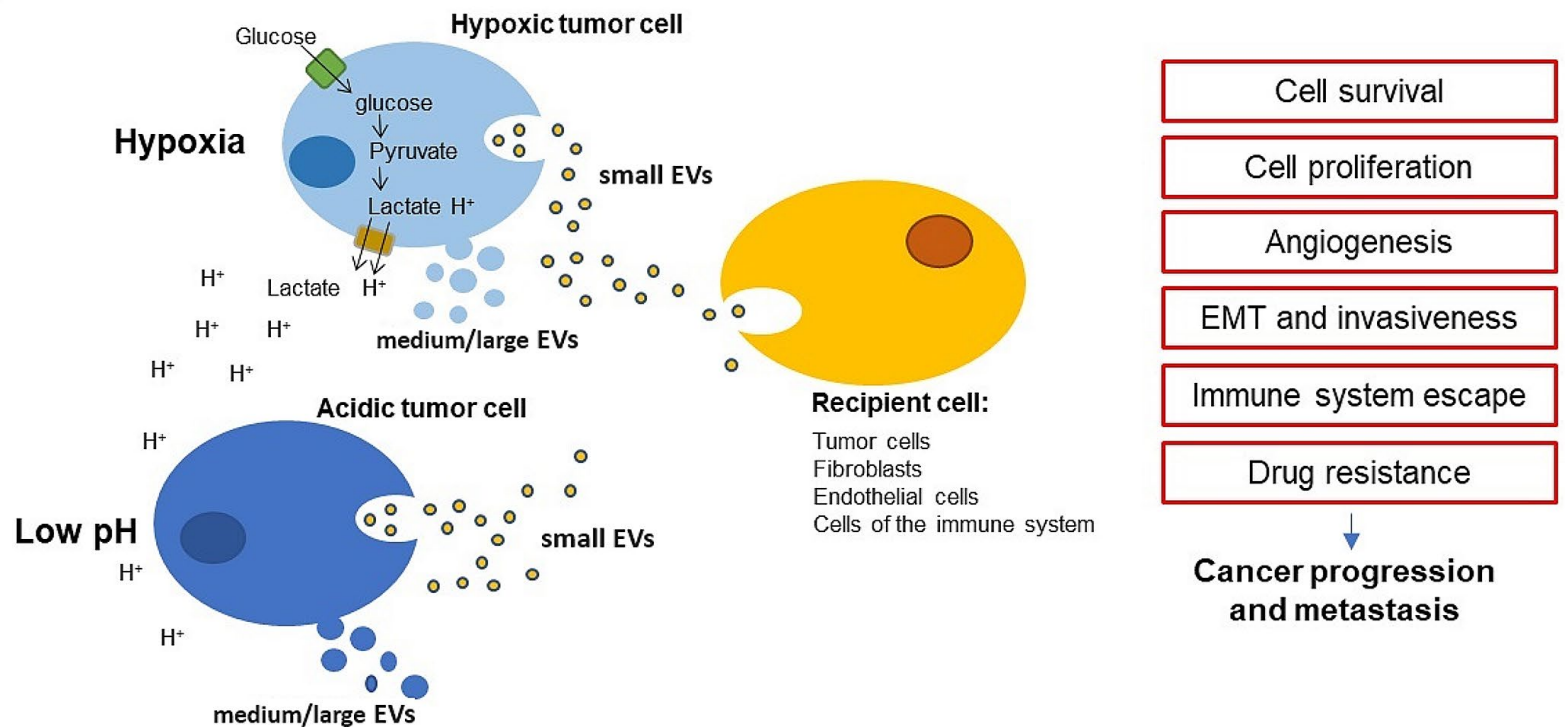
Effects of extracellular vesicles released by hypoxic or acidic tumor cells on different recipient cells

There are several described mechanisms involved in the higher sEV secretion under hypoxia (see Fig. 2) among which the principal one is considered HIF1α signaling [61, 62], which regulates the expression of numerous genes involved in cellular adaptation to low oxygen levels, including genes associated with EV biogenesis, cargo loading, and release, such as PKM2 [75]. Hypoxia can also lead to the upregulation of Rab-GPTases, such as Rab27a and Rab27b [76], which are essential for the formation and trafficking of multivesicular bodies, the precursors of sEV [77, 78]. Other proposed mechanisms playing a role in EV biogenesis under hypoxia are the synthesis of ceramide from sphingomyelin [34], the increase in intracellular calcium levels [79], the regulation of ESCRT machinery, tetraspanins [80] and oxidative stress [81, 82]. Through the increased amount of secreted EVs, low oxygen facilitates tumor extracellular communication at a distance; EVs released into the extracellular environment can be taken up by other cells (see Fig. 3), in which, through the release of their contents, they induce functional responses (see Table 1).

#### Hypoxic EVs and tumor cell survival

It has been described that EVs released by hypoxic tumor cells are capable of increasing cell survival. Through EVs, hypoxic tumor cells induce hypoxia tolerance mechanisms in surrounding cells, which acquire a more hypoxia-tolerant phenotype, improving their survival and tumor malignancy [83]. EVs derived from hypoxic tumor cells are often enriched in HIF-1α-dependent miRNAs, which play a key role in the acquisition of hypoxia tolerance, inducing HIF1α stabilization and angiogenesis, influencing autophagy, or targeting apoptosis. Some examples of these hypoxia-activated miRNAs are represented by miR-21, which, through the activation of Akt and ERK, is involved in resistance to apoptosis, angiogenesis and migration [84–86]; miR-23a, which reduces hypoxia-induced cell death targeting some components involved in apoptosis, such as BH3 interacting domain death agonist (BID), caspase-7 and NIX/BNIP3L [87]; mir-494 which upregulates HIF-1α expression through activating the PI3K/Akt pathway, protecting against hypoxia-induced apoptosis [88]. Zeng et al. recently described a hypoxic vesicular circular RNA (circRNA)-mediated mechanism of conferred resistance in pancreatic cancer (PC) cells: CircZNF91, overexpressed in hypoxic sEVs, can be transmitted to normoxic pancreatic cancer cells, enhancing the stability of HIF1α and leading to chemoresistance of normoxic pancreatic cancer cells [89]. Screening miRNA profiles of mouse plasma sEVs and cultured pancreatic stellate cells (PSCs) allowed researchers to identify miR-4465 and miR-616-3p as principal hypoxia-induced vesicular miRNAs promoting PSC cell proliferation, migration, and invasion, thus contributing to PSC progression and metastasis [90]. miR-361-3p was reported to be significantly elevated in sEVs derived from hypoxic colorectal cancer (CRC) cells, and when transferred to normoxic CRC cells, it facilitates cell growth and suppresses cell apoptosis, by targeting TNF receptor-associated factor 3 and consequently activating the noncanonical NF-κB pathway [91]. Panigrahi et al. [92] have reported that sEV secretion under hypoxia offers a survival advantage to prostate cancer cells, probably through the removal of certain metabolites such as lactic acid and inhibition of sEV secretion significantly inhibited the growth of cancer cells.

#### Hypoxic EVs and angiogenesis

Many studies report that EVs derived from hypoxic tumor cells elicit a greater angiogenic activity from vascular endothelial cells compared to EVs derived from normoxic cells. Muz et al. argue that EV release stimulated by hypoxia is a key mechanism capable of promoting cancer survival and progression by stimulating new blood vessel formation [58] through the promotion of endothelial cell proliferation and migration. The suppression of sEV secretion inhibits these effects and impairs tumor growth [93]. Indeed, different non-coding RNA delivered by hypoxic sEVs have been demonstrated to act as potent inducers of angiogenesis in vivo and in vitro. For example, sEVs derived from hypoxic lung cancer cells contain miR-23a, which can increase angiogenesis and vascular permeability by targeting prolyl hydroxylase 1 and 2 (PHD1 and 2), leading to the accumulation of hypoxia-inducible factor-1 α (HIF-1 α) in endothelial cells, and tight junction protein ZO-1 [94]; Hypoxia-induced secretion of sEVs containing miR-494 from non-small cell lung cancer promotes angiogenesis by downregulation of PTEN and activation of the Akt/eNOS pathway in endothelial cells [95]; sEVs derived from hypoxic leukemia cells enhance the tube formation in human umbilical vein endothelial cells (HUVECs) via miR-210 [96]. Also, tumor-derived hypoxic vesicular miR-30b-5p [97] and miR-182-5p [98], can enhance tube formation when taken up by HUVECs, promoting respectively gap junction protein GJA1 downregulation or KLF2 and KLF4 suppression and VEGFR accumulation. In multiple myeloma, miR-135b was found upregulated in cancer-derived sEVs under hypoxia and was able to enhance angiogenesis in vitro and in vivo via the HIF-FIH signaling pathway [99]. In addition to miRNA, proteins, and mRNA contained in hypoxic EV significantly increase angiogenesis and endothelial migration. Some examples are represented by EV-associated CAIX [100], glypican-3 (GPC3) [101], protein-lysine 6-oxidase (LOX), thrombospondin-1 (TSP1), vascular-derived endothelial factor (VEGF) and a disintegrin and metalloproteinase with thrombospondin motifs 1 (ADAMTS1) [102]. Kucharzewska et al. [103] showed that hypoxic glioma cells produce EVs containing various mRNAs and proteins such as IL-8, PDGFs, caveolin 1, matrix metalloproteinases, and LOX, which modulate endothelial cell phenotype, inducing angiogenesis ex vivo and in vitro. Huang and Feng [93] demonstrated that hypoxia, in a HIF1-dependent manner, induces the secretion by CRC cells of sEVs containing Wnt4a, which increases β-catenin nuclear translocation in endothelial cells leading to angiogenesis and cancer progression. They also found that suppression of sEV secretion through RAB27a knockdown in CRC cells inhibited sEV-induced proliferation and migration of endothelial cells. Guo et al. [104] reported that sEVs derived from hypoxic PC cells expressed a high level of long noncoding RNA (lncRNA) UCA1, which promotes angiogenesis and tumor growth via the miR-96-5p/AMOTL2/ERK1/2 axis. A recent study demonstrated that hypoxic sEVs derived from epithelial ovarian cancer cells induce macrophage M2 phenotype polarization through miR-940 expression [105]. Subsequently, M2 macrophages can secrete angiogenic factors (ANGPT2, IL-8, MMP9, PF4, and TIMP-1) inducing angiogenesis and promoting cancer progression [106]. Several in vitro and in vivo studies have also shown the pro-angiogenic capacity of EVs derived from MSC stimulated by hypoxia [107–109]. In particular, EVs from hypoxic adipose mesenchymal stem cells (ADSCs) were shown to be able to promote the proliferation, migration, and tube-formation capability of HUVECs through VEGF/VEGF-R signaling [109].

#### Hypoxic EVs, EMT, and cell invasion

It has been observed that sEVs produced under hypoxic conditions can stimulate the migration and invasion ability of tumor cells [61, 110]. Hypoxic sEVs contain several miRNAs such as miR-1246, miR-10b-5p [111], miR-4465, miR-616-3p [90], miR-21 and miR-1273f [112], miR-410-3p [113] that have been demonstrated to promote migration and invasion when transferred to normoxic cells. In particular, vesicular miR21, upregulated under hypoxia in oral squamous cell carcinoma cells [84] and in head and neck squamous cell carcinoma (HNSCC) cells [114] in HIF-1α and HIF-2α–dependent ways, was reported to be able to downregulate a pool of genes (PDCD4, PTEN, E-Cadherin) and induce EMT of the target normoxic cells, while miR-1273f, upregulated by hypoxia in EVs derived from hepatocellular carcinoma cells (HCC), promotes proliferation, migration, EMT and invasiveness in normoxic cells by targeting LHX6 and subsequently activating Wnt/β-catenin signaling pathway [112]. Hypoxia, through HIF-1α-driven downregulation of STX11 and SYT7, was also found to promote HCC to secrete sEVs containing GPC3, which significantly contributes to inducing cell migration and EMT, as well as tumor growth in mice [101]. Very recently, Duan et al. [115] demonstrated that vesicular miR-5100 secreted from hypoxic HNSCC cells improves the invasion of tumor cells in vitro and contributes in a HIF1α-dependent manner to the activation of fibroblasts, further promoting the metastasis cascade in HNSCC.

Zhang et al. [116] reported that the transfer of the vesicular miR-193a-3p, miR-210-3p, and miR-5100 from hypoxic bone marrow-derived mesenchymal stem cells (BMSCs) to epithelial cancer cells activate STAT3 signaling-induced EMT. The hypoxia-dependent release of miR-410-3p from CRC was described to induce an increase in proliferation, migration, and invasion of normoxic CRC by downregulating PTEN expression [113]. Ramteke et al [110]. showed that hypoxic EVs downregulate E-cadherin levels and increase β-catenin expression in prostatic cancer cells. Other studies found that miR-210-3p, overexpressed in EVs derived from tumor cells [117, 118] is induced by a HIF1-dependent mechanism and it promotes EMT and invasiveness by upregulating TGF-β [119]. Hypoxia has also been described to be capable of inducing CAF-derived secretion of EVs promoting cancer cell invasion of breast cancer cells, through the autophagy-associated GPR64 [72].

#### Hypoxic EVs and immune response

Hypoxia-induced sEVs play critical roles also modulating immune responses. TAMs are among the most common immune-related stromal cells in the TME. Recent studies, revealed that hypoxic sEVs, derived from epithelial ovarian cancer cells, deliver miR-21–3p, miR-125 b-5p, miR-181 d-5p [120], and miR1225-5p [121], inducing macrophage polarization to an M2-like phenotype. Macrophage polarization into M1 and M2 phenotype in tumors represents a crucial aspect of tumor progression, and in particular, the M2 phenotype is endowed with a tumor-promoting ability involving immune depression, angiogenesis, and stromal activation accelerating aggressiveness, invasion, and metastatic dissemination [122]. The same effect on macrophages polarization was described by Park et al. [123], who observed that sEVs produced by hypoxic tumor cells are highly enriched in immunomodulatory proteins and chemokines such as CSF-1, CCL2, FTH, FTL, and TGFβ, while Qian et al. [124] and Xu et al. [125] found that hypoxic glioma-derived sEVs markedly induce M2 macrophage polarization through miR-1246 and miR155-3p.

Other studies described the effect of EVs derived from hypoxic tumors on NK and T cell function. Berchem et al. [126] showed that NK cells treated with normoxic or hypoxic EVs showed different levels of cytotoxicity; in particular, hypoxic EV inhibited NK cell function and IFN-γ production by a mechanism involving TGF-β1 transfer to NK cells and the consequent decrease of the cell surface expression of the activating receptor NKG2D. Ye et al. [127] found that hypoxia increases in nasopharyngeal carcinoma cells the vesicular miR-24-3p, which inhibits T-cell proliferation and Th1 and Th17 differentiation, while inducing the differentiation of regulatory T-cells (Tregs), via repression of targeting fibroblast growth factor 11 (FGF11) in recipient T cells. Also, Rong et al. [128] described the ability of sEVs derived from hypoxic breast cancer cells to suppress T-cell proliferation. The study revealed that this effect was due to TGF-β contained in hypoxic sEVs [128].

It has been shown the transfer of functional RNA contained in EV from cancer cells to myeloid-derived suppressor cells (MDSCs), which are critical mediators of immunosuppression in the TME [129]. Guo et al. [67] demonstrated that hypoxia-dependent expression of miR-10a and miR-21 in glioma sEVs mediate MDSC expansion and polarization by targeting RAR-related orphan receptor alpha (RORA) and PTEN. Moreover, they found that MSDC exposed to these sEVs reduced T-cell proliferation.

Several recent studies investigating the roles of tumor EVs in immune modulation have highlighted that sEVs play a key role in tumor-immune escape through the PD-L1/PD1 axis [130–132]. Li et al. observed that miR-21 contained in sEVs derived from hypoxic oral squamous cell carcinoma (OSCC) increases the PD-L1 expression of MDSCs, thus decreasing the antitumor ability of γδ T cells (through a miR-21/PTEN/PD-L1 regulation axis) [133]. More recently, Liu et al. [134] demonstrated that intermittent hypoxia enhances the function of EVs derived from lung cancer cells to aggravate immunosuppressive status in macrophages, promoting in these cells PD-L1 and HIF-1α expression. Also, the vesicular circEIF3K secreted by hypoxic CAFs was found capable of accelerating tumor progression by regulating miR-214/PD-L1 in the colorectal cancer cells [73].

#### Hypoxic EVs and drug resistance

Many studies indicate that EVs can transfer therapy resistance from resistant tumor cells to sensitive cells by increasing anti-apoptotic signaling and DNA repair or delivering ABC transporters, thus contributing to increased tumor malignancy [135]. For example, a recent study on non-small cell lung cancer (NSCLC) demonstrated that hypoxic NSCLC cell-derived sEVs promote cisplatin resistance in normoxic cells mainly by the effect of vesicular miR-21 in inhibiting the expression of the tumor suppressor phosphatase and tensin homolog PTEN [136]. Yue et al. revealed that vesicular miR-301a released by hypoxic glioblastoma cells promotes radioresistance by increasing the activity of the Wnt/β-catenin signaling pathway [137], while Yin et al. demonstrated that EVs derived from hypoxic glioma stem-like cells confer temozolomide resistance on glioblastoma by delivering miR-30b-3p [138]. Dorayappan et al. showed that hypoxic ovarian cancer cell-derived sEVs were proficient in significantly decreasing dsDNA damage and increasing cell survival in response to cisplatin treatment [66], while Zhu and colleagues found that sEVs from hypoxic macrophages can promote in vitro and in vivo chemoresistance in ovarian cancer cells, through the vesicular miR-223/PTEN-PI3K/AKT pathway [74]. Hypoxic EVs can also cause treatment resistance by inducing metabolic reprogramming in recipient cells. In this regard, a recent study showed that hypoxia-induced sEVs play a key role in lung cancer drug resistance, either directly leading to cisplatin resistance by transferring PKM2 to tumor cells, or indirectly enhancing cisplatin resistance through lactate production by CAFs, due to glycolysis promotion [139]. Another study on pancreatic cancer (PC) found that hypoxia induces PC cells to release circZNF91-enriched sEVs, which facilitate the transition of normoxic PC cells to hypoxic phenotype with increasing glycolysis and significantly promoting Gemcitabine resistance [89]. Another way sEVs regulate drug resistance is by reducing intracellular drug concentrations, either by regulating drug efflux or by drug sequestration. For example, Kalia’s group, which in a previous work described the role of miR-155, a mi-RNA upregulated in hypoxic conditions [140], in conferring chemoresistance to oral squamous cell carcinoma (OSCC) cells via downregulation of the transcription factor FOXO3a [141], demonstrated that the inhibition of the vesicular miR-155 reverses cisplatin resistance by suppressing drug efflux transporter protein expression in cisplatin-resistant tumors [142].

As seen above, extensive knowledge exists regarding the characteristics and activities of EVs released by tumor cells when exposed to a hypoxic microenvironment. However, hypoxia never comes alone; it is often accompanied by a reduction in pH, resulting in a tumor microenvironment in continuous and dynamic change. Therefore, it is necessary to assess how an acidic microenvironment influences the release of microvesicles.

### Effects of acidic EVs

Extracellular acidosis in tumors is associated with a poor prognosis [143, 144], resistance to therapy [145, 146] and immunosuppression, inhibiting the tumoricidal activity of cytotoxic lymphocytes and natural killer cells [147], and decreasing the proliferation of T cells [148]. TME acidity also stimulates an increased mutation rate [149] contributing to the acquisition of an aggressive phenotype by tumor cells. Several studies have shown that TME acidity induces the epithelial-to-mesenchymal transition (EMT) phenotype associated with the release of proteolytic enzymes, driving tumor cell invasion into host tissues and secondary organ colonization [4, 150]. Moreover, acidosis promotes proangiogenic signaling and vasculogenic mimicry, supporting the possible demand for nutrients and oxygen [151, 152]. Our group reported that acidosis supports *anoikis* resistance [153] and drives a stem-like phenotype in melanoma cells [154]. Moreover, we found that a decreased extracellular pH causes a metabolic adaptation in tumor cells shifting their metabolism from glycolysis to OXPhos [155], as it was reported towards glutamine and fatty acid metabolism [156].

Recent reports show that an acidic pH 6.5 TME increases EV release and uptake by human cancer cells, independently of their histotype, compared to a pH 7.4 TME (Fig. 2), also contributing to tumor progression [157–160]. In particular, Parolini et al. [159] reported that EVs released at low pH by melanoma cells were characterized by high membrane rigidity and enrichment in sphingomyelin/ganglioside GM3 (N-acetylneuraminylgalactosylglucosylceramide) content, which promoted an increase of EV uptake through a fusion mechanism. Consistent with these observations, first Parolini et al. [159] and then Logozzi et al [158]. demonstrated that by counteracting the TME low pH with either buffering or Proton Pump Inhibitors (PPI) significantly reduces sEV release and uptake by tumor cells, suggesting that the inhibition of extracellular acidification may interfere with sEV trafficking within a tumor mass.

More recent studies show that extracellular acidosis stimulates an increase in protein content and Zeta potential (an important physicochemical parameter that influences the stability of the particles) in tumoral EVs, contributing to EV-based cell-cell communication. [160–162].

The proteomic analysis of acidic sEVs performed by Boussadia’s lab disclosed the presence of an increased expression of proteins responsible for cell motility, trans-endothelial migration, invasion, and angiogenesis [163], confirming the primary acidic sEV role in cancer progression (see Fig. 3; Table 1). A meta-analysis study of acidic sEV proteins made by Prognoscan, identified a list of genes (HRAS, GANAB, CFL2, HSP90B1, HSP90AB1, GSN, HSPA1L, NRAS, HSPA5, TIMP3, HYOU1) significantly related to a poor prognosis of melanoma patients [163]. Taraboletti et al. [164] showed that an acidic environment alters the membrane integrity of EVs and promotes the release of intraluminal VEGF-A, sustaining endothelial cell motility and angiogenesis. Other studies provided evidence that sEVs released by acidic tumor cells contain high levels of tumor-associated molecules, that they can transfer to tumor cells that do not express these molecules, such as caveolin-1 [159] and mir-214 [165] in melanoma cells, carbonic anhydrase (CA) IX in prostate cancer cells [166], miR-21 and miR-10b in hepatocellular carcinoma cells [167].

Therefore, acidic tumor cells produce a high amount of EVs, able to affect the behavior of neighboring non-acidic tumor cells through the transfer of critical vesicular proteins.

Considering that TME low pH was reported to affect cell resistance to cytotoxic drugs, Federici et al. showed that Cisplatin uptake by human tumor cells was markedly reduced by acidosis and this effect was mediated by sequestration and elimination of chemotherapeutic drugs by acidic sEVs [157]. Indeed, acidic sEVs rapidly trap a greater amount of Cisplatin, preventing CisPt activation, and allowing tumor cells to avoid intracellular drug accumulation. Moreover, pre-treatment with PPI induced at the same time a marked reduction of sEV release and cisplatin content in sEVs, but an increased tumor cell content of the cytotoxic drug [157].

Although knowledge regarding the characteristics of EVs released by tumor cells in an acidic microenvironment is more limited compared to that of hypoxia, it remains of particular interest. We believe that a comprehensive understanding of how tumor cells adapt to both hypoxic and acidic tumor microenvironments is necessary for the discovery of new therapeutic targets.

**Table 1:** Summary of content and relative effect on cancer progression of hypoxic or acidic extracellular vesicles

	Effect	Vesicle contents	
Hypoxia	Survival	miR-21 miR-23a miR-361-3p	miR-4465 miR-616-3p circ-ZNF91
	Angiogenesis	miR-23a miR-30b-5p miR-182-5p miR-210 miR-494 miR-135b miR-940 CAIX LOX	GPC3 VEGF ADAMTS1 TSP1 IL-8 PDGF Cav1 WNT4a lncRNA UCA1
	EMT and Invasiveness	miR-1246 miR-10b-5p miR-4465 mir-616-3p miR-21 miR-1273f	miR-410-3f miR-193-3p miR-210-3p miR-5100 miR-1255-5p GPC3
	Immune escape	miR-21 miR-125b-5p miR-181d-5p miR-24-3p miR-10a miR-21-3p miR-1246 miR1225-5p	miR155-3p circEIF3K CSF1 CCL2 FTH FTL TGFb
	Drug resistance	miR-21 miR-301a miR-155 miR-223	miR-30b-3p PKM2 circZNF91
Acidosis	Cell Proliferation, migration, invasion	miR-21	miR-10b
	Macrofage activation	miR-214	
	Angiogenesis	CAIX Cav1	VEGFA

## EV clinical application

Tumor biopsy and histological examination although represent fundamental instruments for solid tumor diagnosis during the different stages of malignancy, often it is very hard to perform due to the dramatic health condition of the tumor-bearing patients. Thus, to perform precision medicine, emerging approaches predicting more exactly the treatment strategies needed for a particular patient are to be investigated. Among the several strategies, liquid biopsy applications to assess circulating tumor DNA [168, 169], circulating tumor cells [169], and/or EVs or sEVs [170], seem to possess the ability to play a new and critical role.

EVs are distinctive because of their content in DNA, RNA, and proteins but also many different molecular conjugates. This aspect makes EVs of real clinical utility, monitoring the dynamic aspects of tumor growth and progression in real-time and with very little discomfort for the patients, as liquid biopsy involves. This is particularly true, considering that the optimal tumor phenotype for cell growth and invasion, acquired through successful adaptation to a changing microenvironment, will dynamically change. Hypoxia and acidosis will inevitably develop in TME of tumors, and adaptations to these microenvironmental features are critical for the transition from in situ to invasive cancer phenotype [171]. There is evidence that the release of specific EVs is increased many folds in cancer patients, with hypoxia and acidity being the major determinants inducing this increase by human cancer cells [172]. Hypoxic and acid EVs can thus play an important role in diagnosis, prognosis, and monitoring of cancer. In effect, the quality of the vesicular contents has been shown to vary at the different moments of tumor life such as during local invasion, metastasis, and treatment. For example, circulating EVs diminish following tumor resection [173], whereas they are increased in response to chemotherapy [174]. Further, hypoxic/acidic EVs may contain protein and RNA biomarkers associated with distant dissemination and pre-metastatic niche formation, anticipating metastatic disease in patients [175–178]. Thus, EV release by acidic and hypoxic cells, extensively described by Zaira Boussadia et al. in melanoma [173], might have a fundamental role in early diagnosis, allowing timely clinical intervention; indeed imaging techniques can only detect cancer when, although very small in dimension, may already contain cancer cells relatively advanced in their malignancy and even able to metastasize.

The numerous characteristics of EVs may allow a better identification of the molecular changes, that the tumor undergoes, including drug resistance molecular markers, allowing to predict chemotherapy efficacy. This is of particular importance to reduce toxicity and side effects of inadequate drugs. Hu et al. showed that EVs secreted by CAFs promote metastasis and chemotherapy resistance of CRC cells, and the detection and inhibition of the vesicular miR-92a-3p offers a tool for the prediction and treatment of metastatic diffusion and chemotherapy resistance [179]. Chen et al. disclosed that metastatic melanoma patients exhibit a higher level of PD-L1-containing EVs, able to predict response to immune treatment [131]. EVs secreted by hypoxic tumor cells express a higher level of immunomodulatory proteins, resulting in most cases in immunosuppression and tumor progression [180]. However, analysis of tumor extracellular vesicles revealed a profound heterogeneity in size subclass but also in cargo [49] in part due to cell origin and in part due to the disparate isolation techniques. Furthermore, no reliable methods are available to characterize the large quantity of EVs secreted by normal cells. Thus, a complete standardization will be critical to reproduce retrospective as prospective clinical trials to confirm the validity of the putative EV-based biomarkers.

In recent years, EVs, due to their good biocompatibility, stability, and biodistribution, have been reported to be a promising tool for the bio-delivery of therapeutic RNAs, proteins, and other agents. Cargo manipulation suggests that EVs may prove beneficial as anti-cancer drug delivery vehicles [181]. Moreover, it has been demonstrated that anti-cancer drugs loaded into EVs obtain enhanced anti-tumor efficacy compared to free drugs, and reduced toxicity to normal cells [182], probably due to the acidosis of the TME, which increases the EV uptake. This means that EVs are preferentially delivered to tumors rather than surrounding healthy tissue [159]. Therefore, the manipulation of cell culture conditions could probably optimize targeting efficiency and overall drug delivery performance of low-pH reprogrammed EVs [183]. Due to the critical role of EVs in tumor progression, it is natural to hypothesize that reducing their production could be a promising strategy for cancer therapy. In this sense, hypoxia and acidosis become direct targets to reduce the secretion of EV in the surrounding microenvironment. Indeed, as previously described, it was demonstrated that TME alkalinization significantly reduces EV release and uptake by tumor cells [158, 159]. Moreover, Federici et al. found that the PPI treatment of melanoma cells reduced the resistance to cisplatin [157]. An alternative approach could be represented by the inhibition of the acidity/hypoxia-induced mechanisms involved in EV biogenesis (see Fig. 2), such as ceramide synthesis. In effect, Trajkovic et al. demonstrated that the release of EVs was reduced after the inhibition of neutral sphingomyelinases, which are involved in the synthesis of ceramide [34].

## Conclusions/take-home message

In the context of the complex relationship between cancer cells and host cells, EV release plays a critical role. Indeed, any type of human cancer can secrete EVs facilitating both primary tumor progression and pre-metastatic niche. Among the several aspects involved in EV secretion by tumor cells, local hypoxia, and acidosis are the major responsible. Hypoxia and acidosis work together and complement each other for EV release, ensuring the sending at all times suitable messages advantageous for the survival and dissemination of tumor cells. Thus, studying the EV release by tumor cells in their hypoxic and acidic microenvironment represents a tool to understand and predict the dynamic changes occurring in a tumor useful for diagnosis and therapy. It is necessary to investigate the specific characteristics of the EVs released by each tumor histotype to monitor dynamically tumor progression providing a preventive diagnosis and therapy. However, despite these promising conditions, we must remember that it is essential, for the discrimination between tumor EVs and the large vesicular population made by normal cells, to set up a reliable qualitative/quantitative procedure also to use EVs for target delivery of specific drugs.

## Data Availability

No datasets were generated or analysed during the current study.
